# Supplementary material for: A predictive index for health status using species-level gut microbiome profiling
Source: Nat Commun. 2020 Sep 15;11:4635. doi: 10.1038/s41467-020-18476-8 (PMC7492273; doi:10.1038/s41467-020-18476-8)
Supplement: Supplementary file 2 — Supplementary Information [file 41467_2020_18476_MOESM2_ESM.pdf]

Supplementary Information

for

A Predictive Index for Health Status Using Species-level Gut Microbiome Profiling

Gupta *et al.*

Table of Contents

SUPPLEMENTARY FIGURES 3

Supplementary Figure 1. 3

Supplementary Figure 2. 4

Supplementary Figure 3. 5

Supplementary Figure 4. 6

Supplementary Figure 5. 7

Supplementary Figure 6. 8

Supplementary Figure 7. 9

Supplementary Figure 8. 10

Supplementary Figure 9. 11

SUPPLEMENTARY TABLES 12

Supplementary Table 1. 12

Supplementary Table 2. 13

Supplementary Table 3. 14

Supplementary Table 4. 15

Supplementary Table 5. 16

Supplementary Table 6. 17

Supplementary Table 7. 18

Supplementary Table 8. 19

Supplementary Table 9. 20

SUPPLEMENTARY NOTE 21

Supplementary Note 1. 21

Supplementary Note 2. 22

SUPPLEMENTARY METHODS 24

SUPPLEMENTARY FIGURES

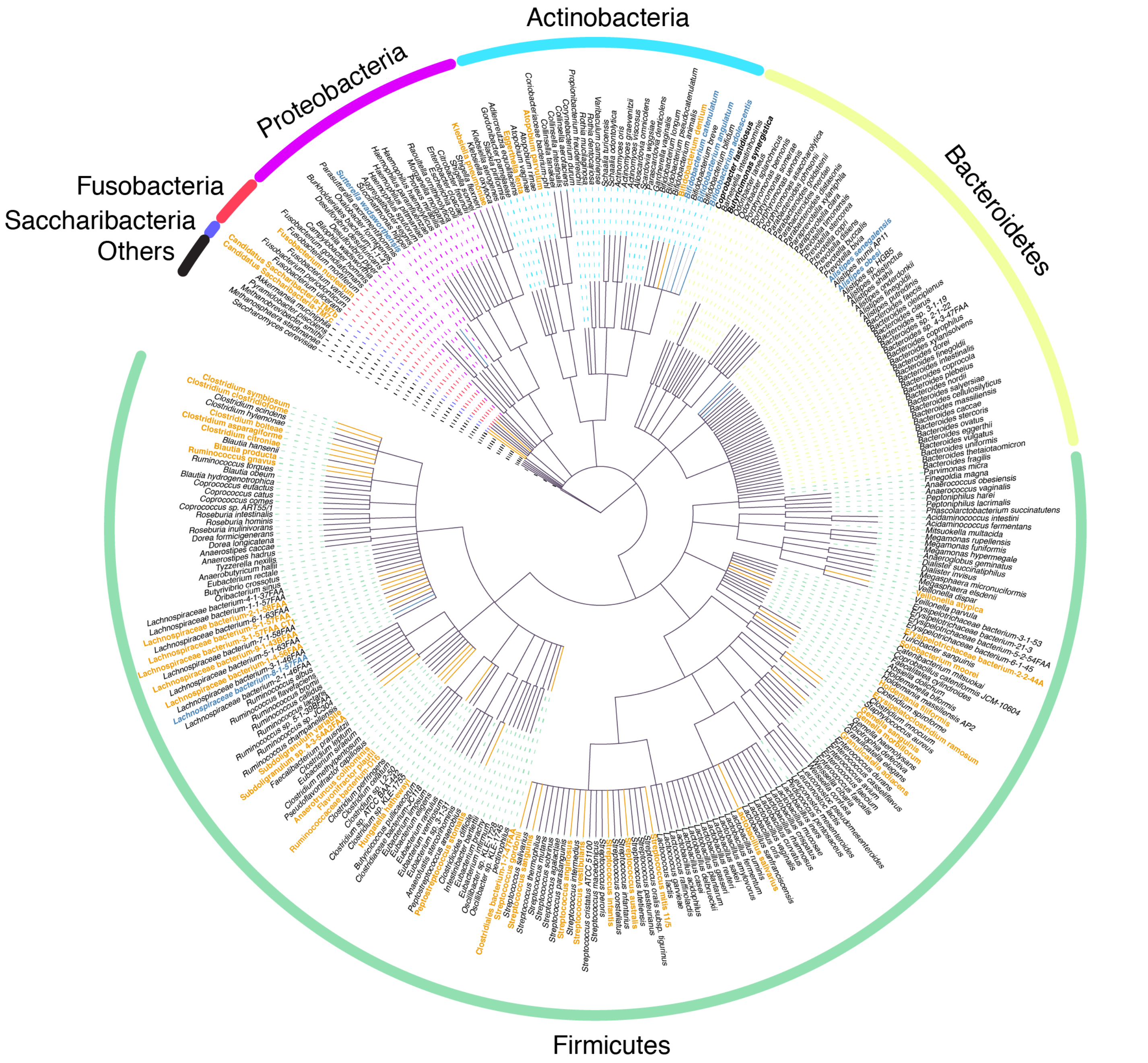

Supplementary Figure 1. A phylogenetic tree showing the evolutionary relationships among 313 microbial species found to be present across 4,347 stool metagenomes. Microbial species comprising the Health-prevalent and Health-scarce groups are shown in blue and orange, respectively. Species are grouped according to their phyla (outer circle labels). 'Others' correspond to the Verrucomicrobia (for *Akkermansia muciniphila*), Synergistetes (for *Pyramidobacter piscolens*), Ascomycota (for *Saccharomyces cerevisiae*), and Euryarchaeota (for *Methanobrevibacter smithii* and *Methanosphaera stadtmanae*) phyla.

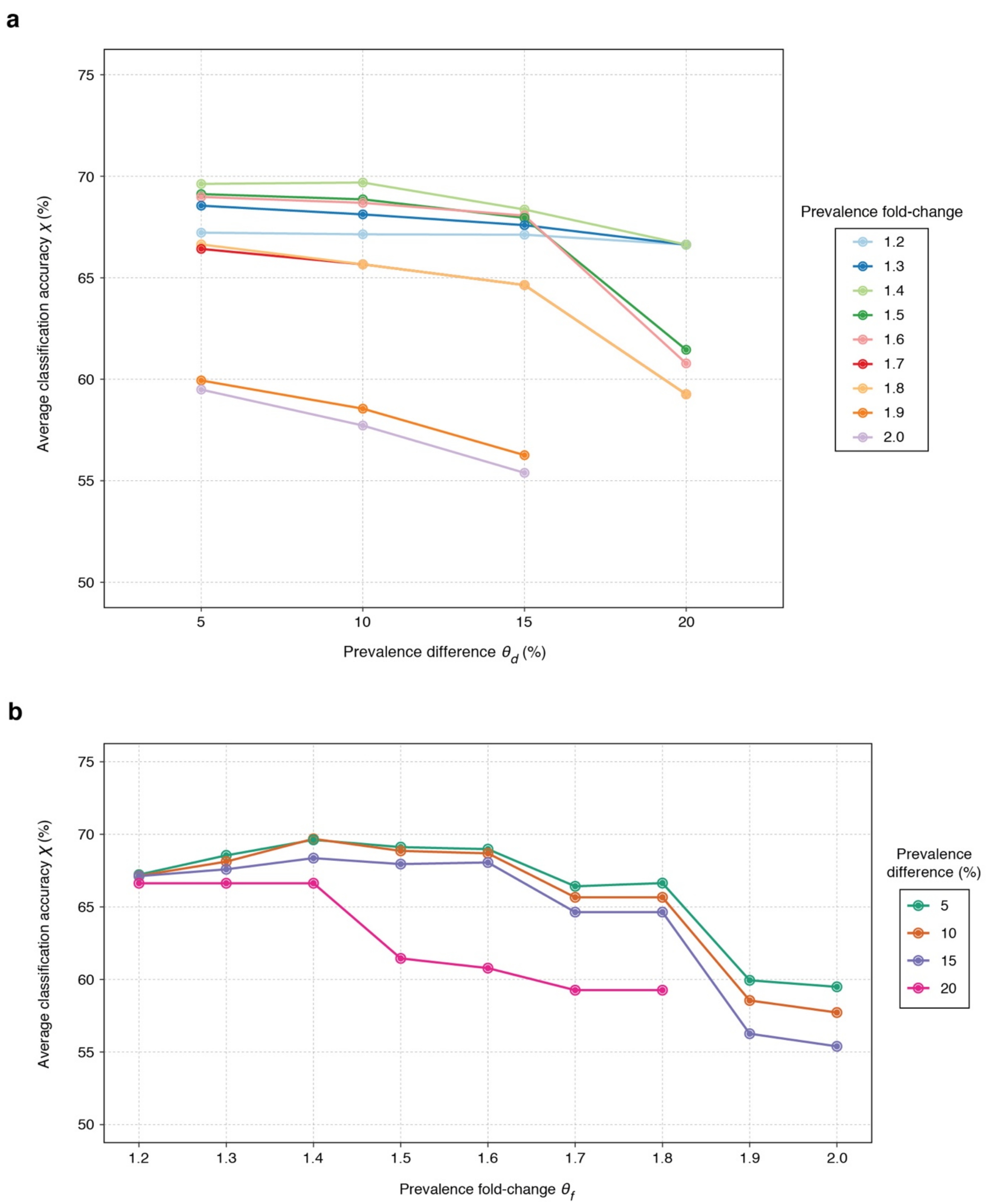

Supplementary Figure 2. Sensitivity analysis of classification performance (i.e., balanced accuracy χ) with respect to a prevalence threshold (θ_f or θ_d). (a) θ_d and χ generally portray an inverse correlation. **(b)** χ displays a very weak but positive correlation for smaller values of θ_f, but then follows an inverse correlation for higher values of θ_f.

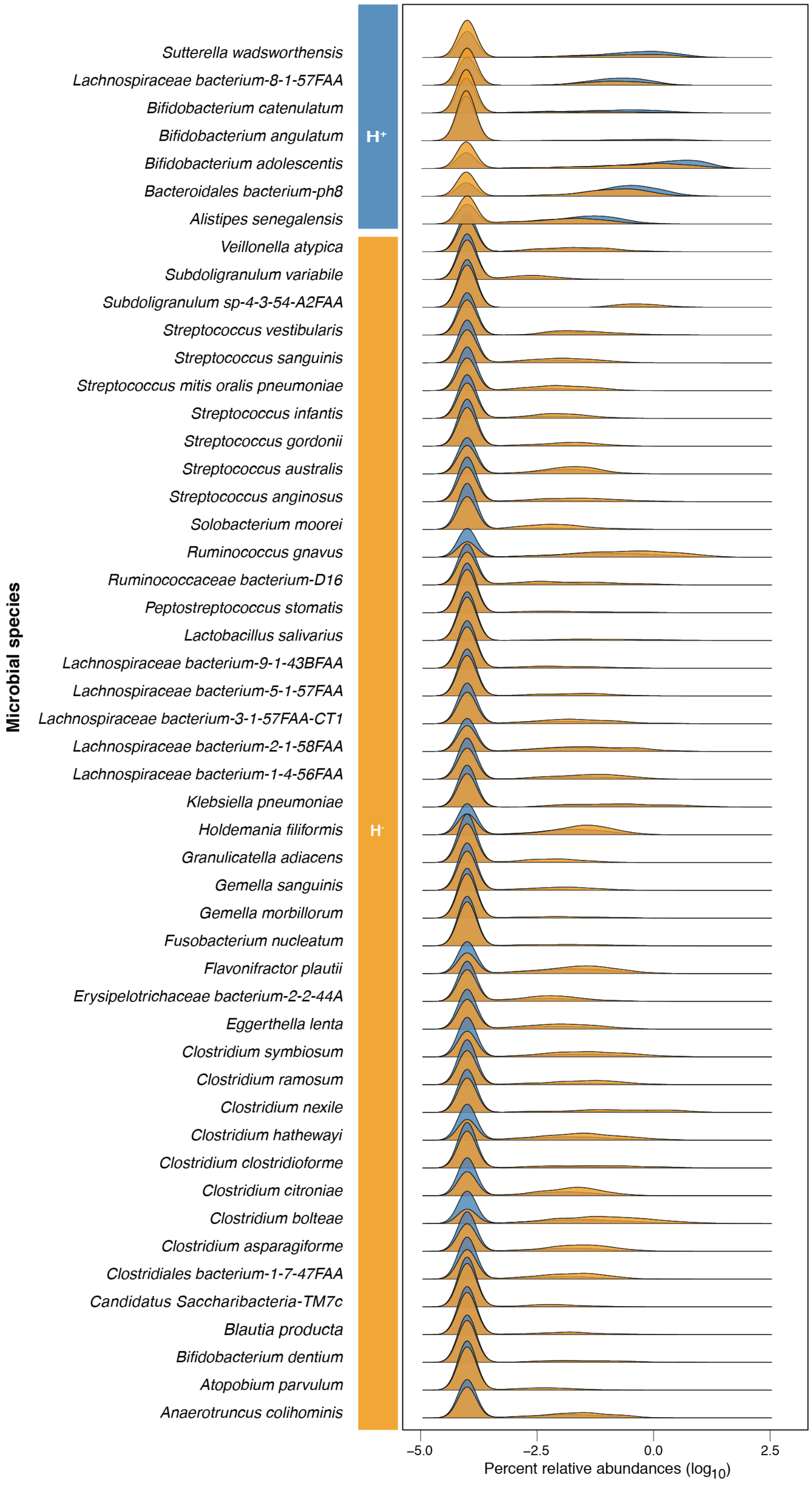

Supplementary Figure 3. Relative abundance distributions of all 50 species in the healthy (blue density plot) and non-healthy (orange density plot) groups. Health-prevalent and Health-scarce species all show higher relative abundance distributions among healthy and non-healthy gut microbiome samples, respectively.

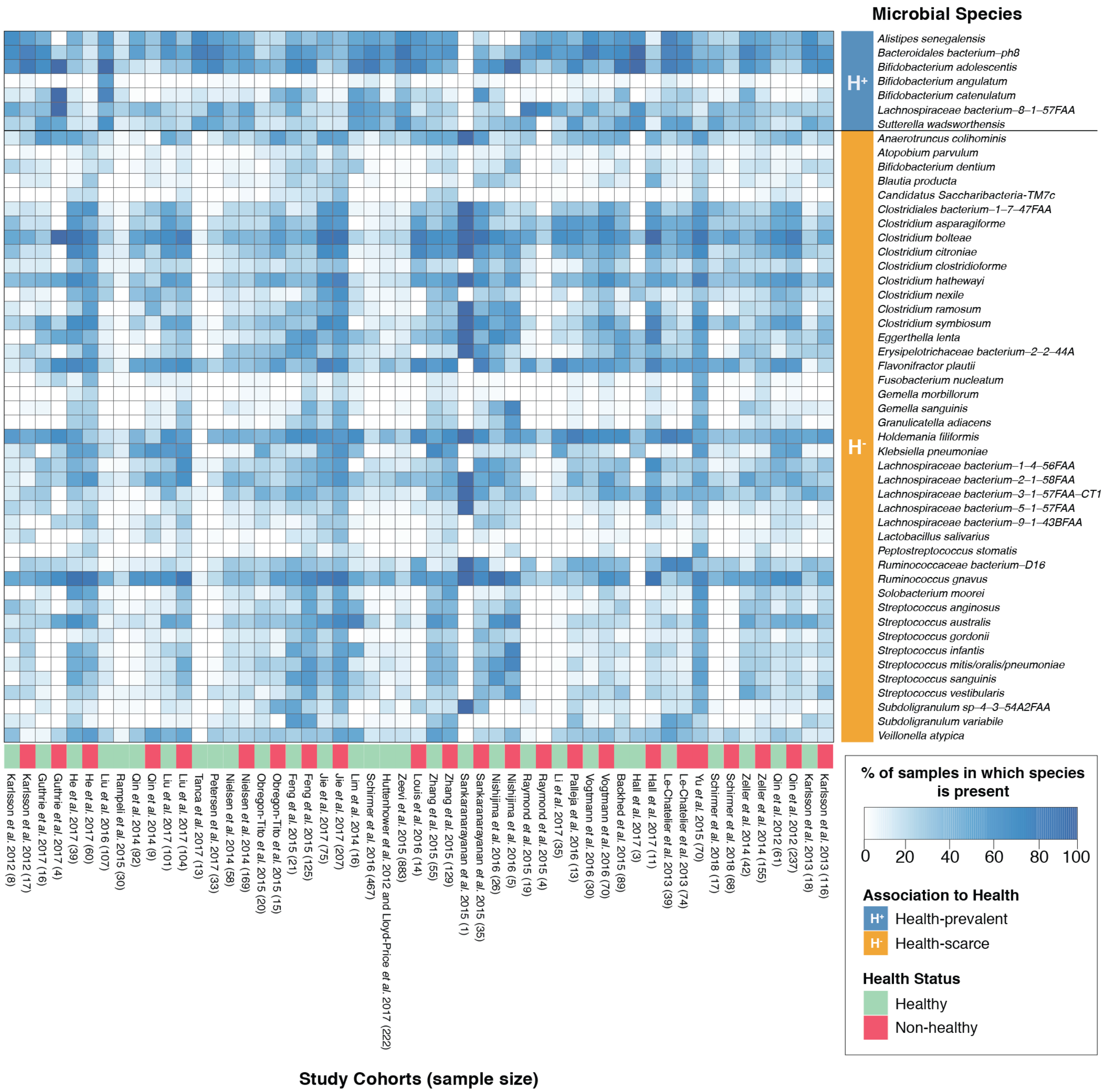

Supplementary Figure 4. Heatmap indicating the prevalence of Health-prevalent and Health-scarce species in the healthy and/or non-healthy cohorts from each of the 34 published studies comprising the stool metagenome meta-dataset.

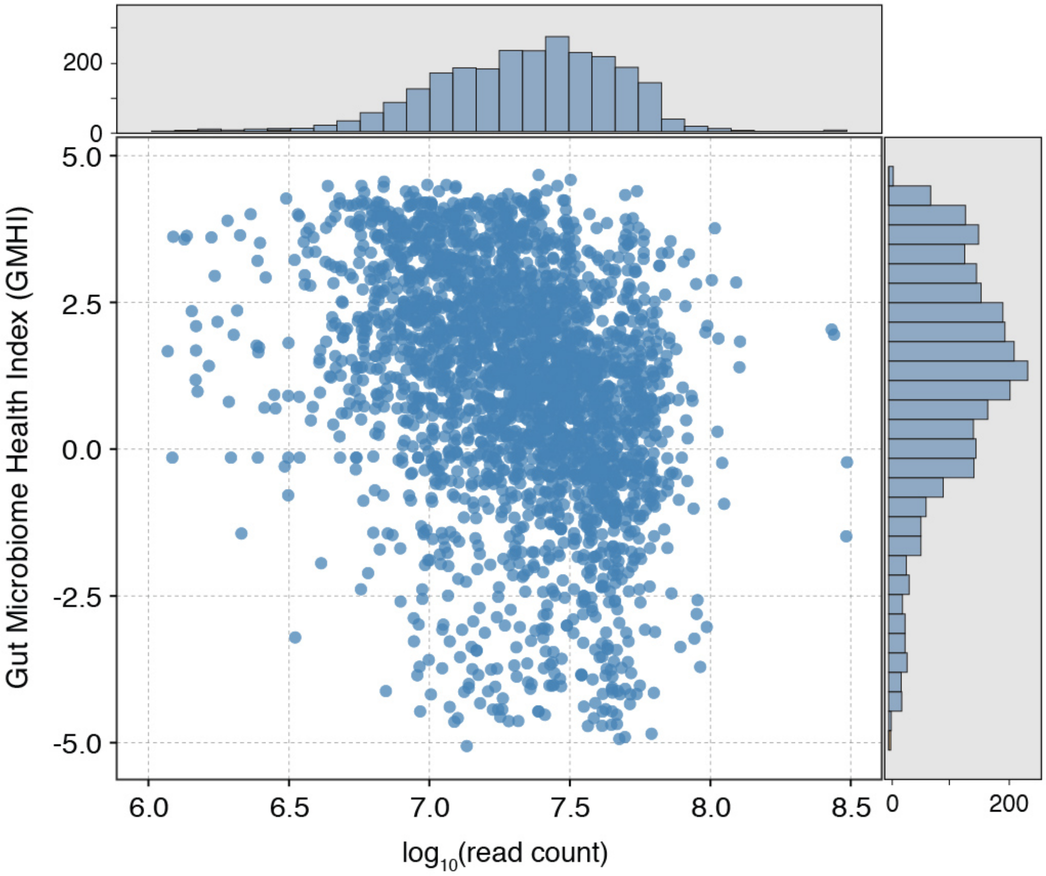

Supplementary Figure 5. Library size (i.e., read count) is not associated with GMHI. Scatter-plot showing the relationship between library size and GMHI for all metagenome samples (n = 4,347). A strong trend between the two parameters was not observed. In addition, mixed-effects linear regression ('lmer' function in the R package 'lme4') was used to create a model for GMHI, wherein model covariates consisted of read count and study of origin (the latter as a random effect to accommodate for inter-study variance). Our model found no significant association between library size and GMHI ($P = 0.45$).

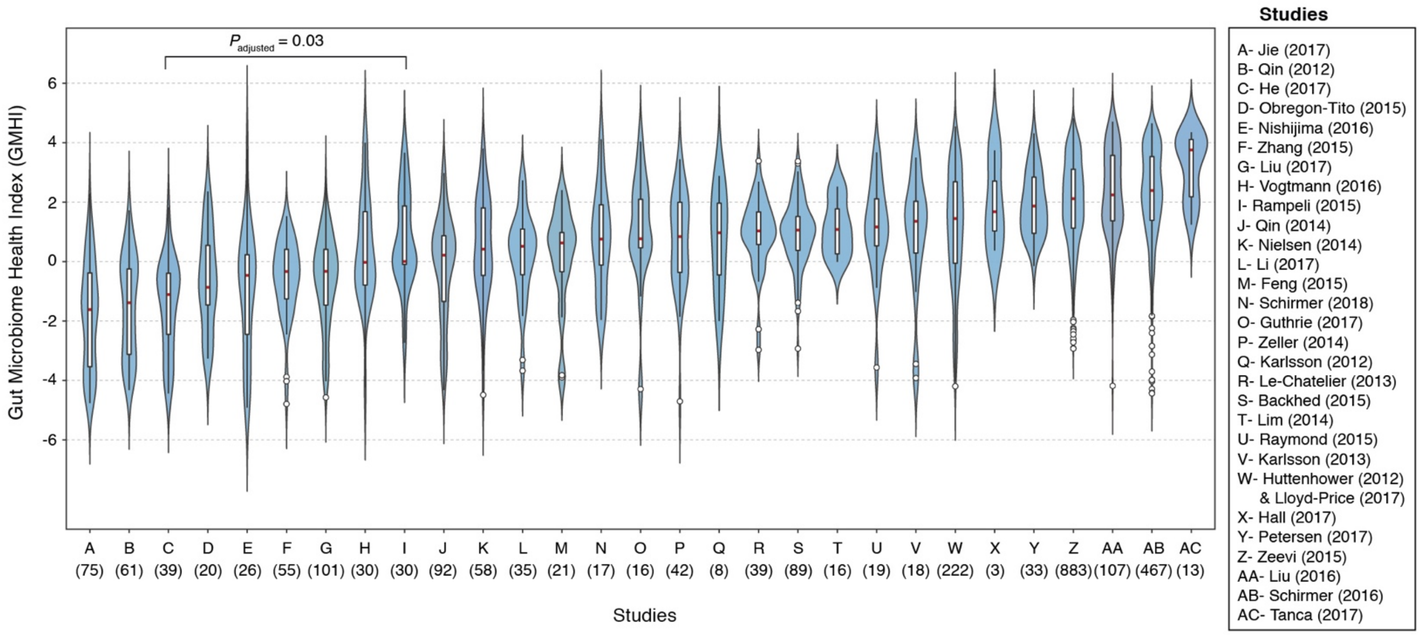

Supplementary Figure 6. Distribution of GMHIs for healthy individuals generally do not vary between studies. Among the 34 studies used in our discovery cohort, i.e., training dataset (**Table 1**), 31 that contain gut microbiome samples from healthy subjects were chosen to investigate whether GMHI distributions from healthy individuals significantly differ between studies. Of these 31, Sankaranarayanan *et al*. was not considered, as it has only a single sample from healthy; in addition, the two HMP1 studies, i.e., Huttenhower *et al*. (HMP1) and Lloyd-Price *et al*. (HMP1-II), were merged. The sample size of each study is shown in the parentheses. A wide variation was observed among the GMHI distributions from study to study. Among all pairwise comparisons between study groups, only one pair of cohorts was found to have distributions significantly different from each other (Dwass-Steel-Critchlow-Fligner test followed by Holm-Bonferroni method to control for family-wise error rate, P_{adj} = 0.03). This shows that, by and large, the distributions of the index for healthy individuals do not vary much between studies. Furthermore, most healthy cohorts (22 of the 29 independent sources) were found to show positive GMHI distributions based on their medians.

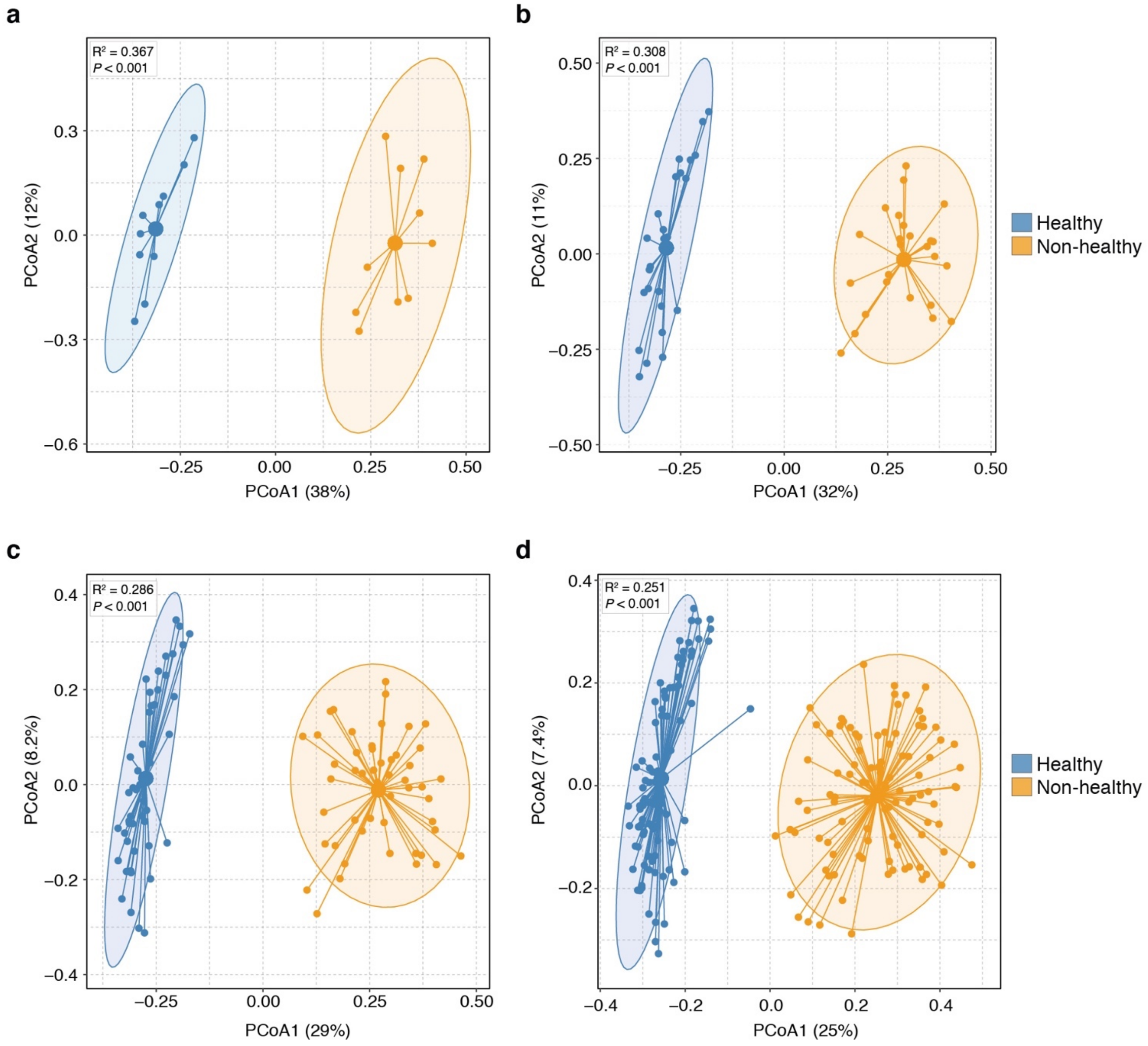

Supplementary Figure 7. Top healthy and non-healthy stool metagenomes, as defined by their GMHIs, show clear separation based on gut microbiome composition. Principal Coordinates Analysis (PCoA) ordination plot based on Bray-Curtis distances for top **(a)** 10; **(b)** 25; **(c)** 50; and **(d)** 100 healthy and non-healthy stool metagenomes samples (identified based on GMHI score) show that healthy and non-healthy groups have significantly different distributions of gut microbiome profiles (PERMANOVA, $P < 0.001$). Large points in the middle of both healthy (blue) and non-healthy (orange) regions depict centroids of all other points, which correspond to stool metagenome samples. Each shaded ellipse represents the 95% confidence region for the centroid of each group. Dispersion of the groups are shown using blue- and orange-colored straight lines between centroid and each sample of its respective group.

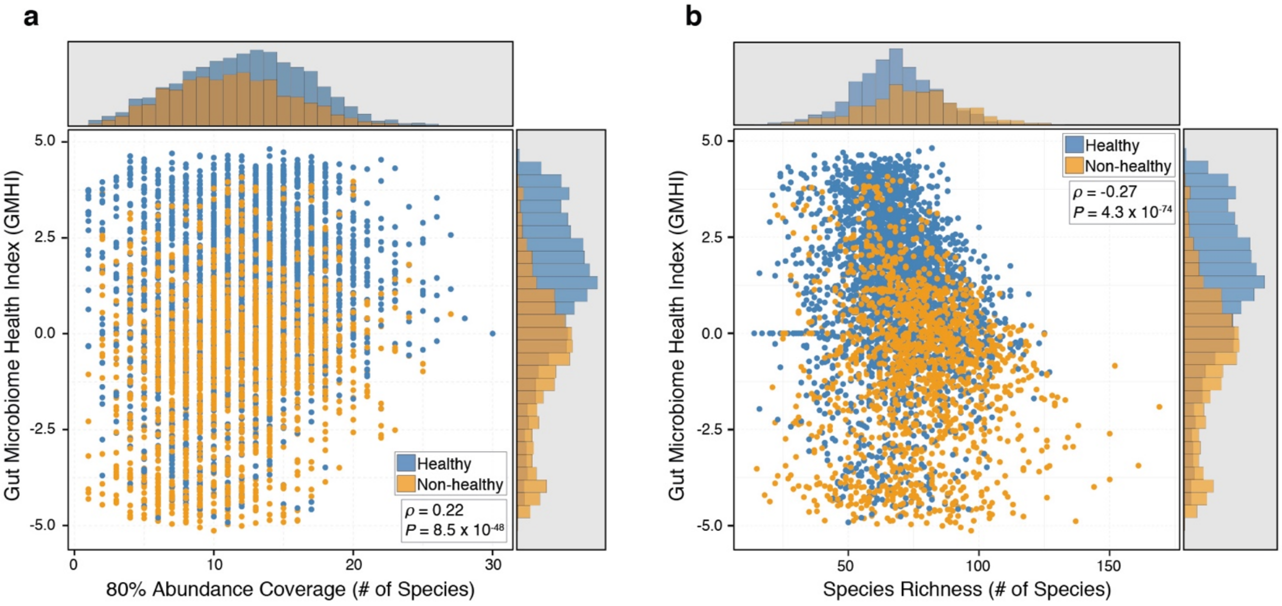

Supplementary Figure 8. GMHI stratifies healthy (n = 2,636) and non-healthy (n = 1,711) groups more strongly than (a) 80% abundance coverage; and (b) species richness. Each point in the scatter-plot corresponds to a sample. Histograms show the distribution of healthy (blue) and non-healthy (orange) samples based on the parameter of each axis. In general, GMHI demonstrates weak correlations with 80% abundance coverage (Spearman's ρ = 0.22, 95% CI: [0.19, 0.25], P = 8.5×10^{-48}) and richness (Spearman's ρ = -0.27, 95% CI: [-0.30, -0.24], P = 4.3×10^{-74}). The P-value (H_0: ρ = 0) was determined by using a t-distribution with n-2 degrees of freedom, where n is the total number of observations.

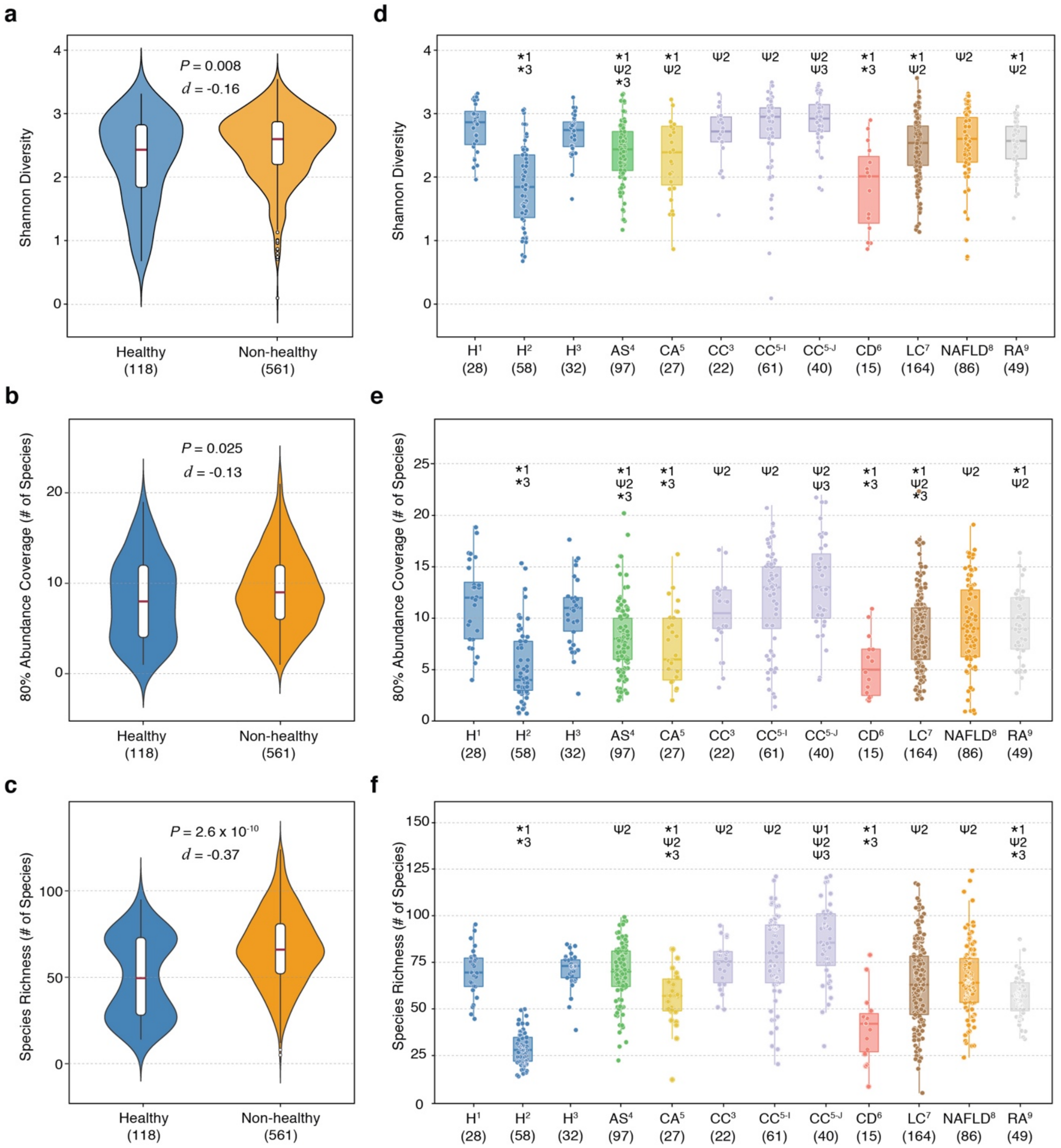

Supplementary Figure 9. Evaluation of other ecological characteristics in distinguishing healthy from non-healthy phenotypes of the validation cohort. (a) Shannon diversity was significantly lower in healthy than in non-healthy individuals (two-sided Mann-Whitney *U* test, $P = 0.008$). **(b)** 80% abundance coverage (two-sided Mann-Whitney *U* test, $P = 0.025$) and **(c)** species richness (two-sided Mann-Whitney *U* test, $P = 2.6\times10^{-10}$) in stool metagenomes were significantly different between the healthy group and non-healthy group. **(d)** Shannon diversity; **(e)** 80% abundance coverage; and **(f)** species richness showed very inconsistent results in distinguishing healthy from non-healthy sub-cohorts. The number in superscript adjacent to phenotype abbreviations corresponds to a particular study used in validation (see **Supplementary Table 5** for study information). Standard box-and-whisker plots (e.g., center line, median; box limits, upper and lower quartiles; whiskers, 1.5× interquartile range; points, samples) are used to depict groups of numerical data. * indicates significantly higher distribution in healthy sub-cohort (two-sided Mann-Whitney *U* test, $P < 0.01$). The number adjacent to * indicates the healthy sub-cohort (H^1, H^2, or H^3) to which the respective sub-cohort was compared. The sample size of each group or cohort is shown in parentheses. AS, ankylosing spondylitis; CA, colorectal adenoma; CC, colorectal cancer; CD, Crohn's disease, H, healthy; LC liver cirrhosis; NAFLD, non-alcoholic fatty liver disease; RA, rheumatoid arthritis.

SUPPLEMENTARY TABLES

Supplementary Table 1. Prevalence thresholds used to select Health-prevalent and Health-scarce species with ensuing classification performances for predicting general health status (i.e., healthy or non-healthy).

Difference[a], $\|P_H - P_N\|$ (%)	Fold-change[b], P_H/P_N or P_N/P_H	# of Health-prevalent Species	# of Health-scarce Species	Balanced Accuracy[c]
10	1.4	7	43	69.7
5	1.4	12	77	69.6
5	1.5	8	75	69.1
5	1.6	5	69	69.0
10	1.5	4	42	68.9
10	1.6	4	39	68.7
5	1.3	16	82	68.6
15	1.4	6	25	68.4
10	1.3	9	45	68.1
15	1.6	3	23	68.1
15	1.5	3	25	68.0
15	1.3	7	27	67.6
5	1.2	27	87	67.2
10	1.2	14	47	67.1
15	1.2	8	27	67.1
5	1.8	4	62	66.6
20	1.2	3	12	66.6
20	1.3	3	12	66.6
20	1.4	3	12	66.6
5	1.7	4	66	66.4
10	1.7	3	36	65.7
10	1.8	3	36	65.7
15	1.7	2	21	64.6
15	1.8	2	21	64.6
20	1.5	1	12	61.5
20	1.6	1	11	60.8
5	1.9	3	59	59.9
5	2	3	54	59.5
20	1.7	1	9	59.3
20	1.8	1	9	59.3
10	1.9	2	34	58.5
10	2	2	30	57.7
15	1.9	1	21	56.3
15	2	1	18	55.4
20	1.9	0	9	N/A
20	2	0	9	N/A

[a]Absolute difference between prevalence of a species in the healthy group (P_H) and that of the same species in the nonhealthy group (P_N). Differences of 5, 10, 15, and 20% were assessed. [b]Ratio of larger value to smaller value. Fold-changes of 1.2 to 2.0, at increments of 0.1, were assessed. [c]As defined by χ in the Results section of this manuscript. Abbreviation: N/A, Not Applicable, as Health-prevalent and/or Health-scarce species are not defined.

Supplementary Table 2. Each feature set (i.e., taxonomic rank or MetaCyc pathways)'s highest balanced accuracy for predicting general health status (i.e., healthy or non-healthy).

Feature Set	Difference[a], $\lvert P_H - P_N \rvert$ (%)	Fold-change[b], P_H/P_N or P_N/P_H	# of Health-prevalent Species	# of Health-scarce Species	Balanced Accuracy[c]
Phylum	5	1.1	2	2	42.1
Class	5	1.1	3	3	60.1
Order	5	1.1	3	6	62.4
Family	10	1.1	2	10	67.2
Genus	5	1.4	3	25	68.2
Species	10	1.4	7	43	69.7
MetaCyc pathways[d]	5	1.1	8	121	59.4

[a]Absolute difference between prevalence of a species in the healthy group (P_H) and that of the same species in the nonhealthy group (P_N). Differences of 5, 10, 15, and 20% were assessed. [b]Ratio of larger value to smaller value. Fold-changes of 1.1 to 2.0, at increments of 0.1, were assessed. [c]As defined by χ in the Results section of this manuscript. [d]MetaCyc metabolic pathway abundances obtained through HUMAnN2 (Franzosa *et al*. Nature Methods (2018), PMCID: PMC6235447).

Supplementary Table 3. Classification accuracy of GMHI in 10-fold cross-validation.

Cross-validation Loop	Difference[a], $\lvert P_H-P_N \rvert$ (%)	Fold-change[b], P_H/P_N or P_N/P_H	# of Health-prevalent Species	# of Health-scarce Species	Accuracy in Healthy Subjects (%)	Accuracy in Non-healthy Subjects (%)	Balanced Accuracy[c]
1	5	1.4	11	77	81.4	67.3	74.3
2	5	1.4	12	75	78.7	64.3	71.5
3	5	1.4	13	79	76.8	59.1	67.9
4	5	1.4	12	79	74.9	61.4	68.2
5	5	1.4	12	78	78.8	56.7	67.8
6	5	1.4	12	78	77.7	64.3	71.0
7	5	1.4	12	77	73.9	63.7	68.8
8	5	1.4	13	77	78.4	56.7	67.6
9	5	1.5	9	77	73.5	62.6	68.0
10	10	1.4	7	40	74.6	67.4	71.0

[a]Absolute difference between prevalence of a species in the healthy group (P_H) and that of the same species in the nonhealthy group (P_N). Differences of 5, 10, 15, and 20% were assessed. [b]Ratio of larger value to smaller value. Fold-changes of 1.2 to 2.0, at increments of 0.1, were assessed. [c]As defined by χ in the Results section of this manuscript.

Supplementary Table 4. Intra-study comparisons among GMHI and other microbiome ecological characteristics in distinguishing healthy and non-healthy groups.

Author (Year)[a]	Healthy (sample #)	Non-healthy[b] (sample #)	*P*-values[c]			
			GMHI	Shannon Diversity	80% Abundance Coverage	Species Richness
Feng (2015)	21	125	6.25E-02	8.41E-01	7.58E-01	3.10E-02
He (2017)	39	60	8.30E-06	7.20E-06	4.70E-06	4.60E-05
Jie (2017)	75	207	4.83E-02	2.16E-01	1.89E-01	8.30E-07
Karlsson (2013)	18	116	7.87E-02	8.94E-01	2.49E-01	4.53E-01
Le Chatelier (2013)	39	74	6.44E-01	3.60E-02	1.50E-02	6.40E-02
Liu (2017)	101	104	3.90E-03	3.17E-01	4.56E-01	4.99E-01
Nielsen (2014)	58	169	6.41E-01	6.98E-01	8.40E-01	3.78E-01
Qin (2012)	61	237	6.81E-01	9.95E-01	6.42E-01	3.96E-01
Schirmer (2018)	17	68	2.60E-03	1.45E-01	2.60E-02	4.06E-01
Vogtmann (2016)	30	70	4.80E-03	7.89E-01	8.80E-01	1.85E-01
Zeller (2014)	42	155	5.00E-04	2.32E-01	1.39E-01	8.18E-01
Zhang (2015)	55	129	3.52E-01	8.61E-01	9.23E-01	5.26E-01

[a]Our criteria for selecting which cohorts to perform intra-study, case-control comparisons was to have both groups to be composed of at least 10 samples, which we deemed as reasonably sufficient sample size; in this case, there were only 12 studies (i.e., cohorts) that satisfied this sample size cut-off. [b]All non-healthy phenotypes were pooled together (when applicable) into a single 'Non-healthy' group. [c]*P*-values (Mann-Whitney *U* test) for each study-specific comparison between healthy and non-healthy groups. *P*-values less than 0.05 are highlighted.

Supplementary Table 5. Independent validation set of human stool metagenomes.

Study #	Author (Year)[a]	Healthy (n)	Unhealthy		Total (n)	Sequencing Platform	Geography (Ethnicity/Race[c])
			Disease[b]	n			
1	Bedarf (2017)	28	-	-	28	Illumina HiSeq 4000	Germany
2	Dhakan (2019)	58	-	-	58	NextSeq 500	India
3	Wirbel (2019)	32	CC	22	54	Illumina HiSeq 2000/4000	Germany
4	Wen (2017)	-	AS	97	97	Illumina HiSeq 2000	China
5	Thomas (2019)	-	CA, CC	CA: 27, CC[d]: 101	128	Illumina HiSeq 2500	Italy & Japan
6	Vaughn (2016)	-	CD	15	15	Illumina HiSeq 2000	USA
7	Qin (2014)	-	LC	164	164	Illumina HiSeq 2000	China
8	Loomba (2017)	-	NAFLD	86	86	Illumina HiSeq 2500	USA (white & hispanic)
9	This study	-	RA	49	49	Illumina HiSeq 3000/4000	USA
	Total	118	-	561	679	-	-

[a]Bibliography provided in **Supplementary Data 4**. [b]AS: Ankylosing Spondylitis; CA: Colorectal Adenoma; CC: Colorectal Cancer; CD: Crohn's Disease; LC: Liver Cirrhosis; NAFLD: Nonalcoholic Fatty Liver Disease; RA: Rheumatoid Arthritis. [c]As provided in the original study. [d]Colorectal cancer samples were from two different cohorts (Italy and Japan); therefore, validation analyses were performed separately.

Supplementary Table 6. Classification accuracy using the Health-prevalent species as class cut-offs.

Dataset Used to Evaluate Classification Performance	Health-prevalent Species Cut-off[a]	Accuracy in Healthy Subjects (%)	Accuracy in Non-healthy Subjects (%)	Balanced Accuracy[b]
Discovery Cohort (4,347 samples)[c]	1	93.5	16.2	54.9
	2	84.0	38.7	61.3
	3	69.2	61.4	65.3
	4	48.5	84.1	66.3
	5	27.3	94.9	61.1
	6	10.2	98.8	54.5
	7	2.7	100.0	51.4
Validation Cohort (679 samples)[d]	4[e]	33.9	84.7	59.3

[a]A metagenome sample was classified as healthy if at least this number of Health-prevalent species were present; else, classified as non-healthy. There are a total of seven Health-prevalent species. [b]As defined by χ in the Results section of this manuscript. [c]Healthy: 2,636 samples; Non-healthy: 1,711 samples. [d]Healthy: 118 samples; Non-healthy: 561 samples. [e]Health-prevalent species cut-off that resulted in the highest balanced accuracy in the discovery cohort.

Supplementary Table 7. Classification performances using Shannon diversity as class cut-offs.

Dataset Used to Evaluate Classification Performance	Shannon Diversity Cut-off[a]	Accuracy in Healthy Subjects (%)	Accuracy in Non-healthy Subjects (%)	Balanced Accuracy[b]
Discovery Cohort (4,347 samples)[c]	Q1	77.3	28.5	52.9
	Median	52.9	54.4	53.6
	Q3	27.8	79.3	53.5
Validation Cohort (679 samples)[d]	Q1	43.2	42.8	43.0
	Median[e]	21.2	72.9	47.0
	Q3	6.8	90.2	48.5

[a]Shannon diversity cut-offs were defined from all 4,347 samples of the discovery cohort, i.e., training dataset. A metagenome sample was classified as healthy if its Shannon diversity was equal to or greater than this cut-off; else, classified as non-healthy. [b]As defined by χ in the Results section of this manuscript. [c]Healthy: 2,636 samples; Non-healthy: 1,711 samples. [d]Healthy: 118 samples; Non-healthy: 561 samples. Abbreviations: Q1, the 1st quartile (=2.50); median (=2.85); Q3, the 3rd quartile (=3.11). [e]Shannon diversity cut-off that resulted in the highest classification accuracy in the discovery cohort.

Supplementary Table 8. All accuracies for classifying healthy vs. non-healthy by the various classifiers reported in this study.

Classifier	Performance on Discovery Cohort (4,347 samples)		Performance on Validation Cohort (679 samples)		
	Methodology to Evaluate Classification Performance	Accuracy (%)	Balanced Accuracy (%)	Accuracy in Healthy (%)	Accuracy in Non-healthy (%)
GMHI (species)	Balanced accuracy	69.7	73.7	77.1	70.2
GMHI (genus)	Balanced accuracy	68.2	-	-	-
GMHI (family)	Balanced accuracy	67.2	-	-	-
GMHI (order)	Balanced accuracy	62.4	-	-	-
GMHI (class)	Balanced accuracy	60.1	-	-	-
GMHI (phylum)	Balanced accuracy	42.1	-	-	-
GMHI (MetaCyc pathways)	Balanced accuracy	59.4	-	-	-
GMHI (species)	10-fold cross-validation[a]	69.6	-	-	-
Health-prevalent species	Balanced accuracy	66.3	59.3	-	-
Shannon diversity	Balanced accuracy	53.6	47.0	-	-
Random Forests	Balanced accuracy	98.5	52.3	-	-

[a]Balanced accuracy was found in each cross-validation loop. Accuracy (%) is then reported as the mean of the ten balanced accuracies.
'-': Not reported.

Supplementary Table 9. Cliff's Delta effect-sizes for all pairwise comparisons in gut microbiome characteristics between healthy and non-healthy sub-cohorts.

	**Cliff's Delta (*d*)[a]**											
	**GMHI**			**Shannon Diversity**			**80% Abundance Coverage**			**Species Richness**		
Cohorts[b]	H[1] (28)	H[2] (58)	H[3] (32)	H[1] (28)	H[2] (58)	H[3] (32)	H[1] (28)	H[2] (58)	H[3] (32)	H[1] (28)	H[2] (58)	H[3] (32)
AS[4] (97)	0.49	0.51	0.60	0.48	-0.50	0.39	0.50	-0.45	0.46	-0.01	-0.95	0.05
CA[5] (27)	0.17	0.24	0.32	0.53	-0.38	0.39	0.56	-0.34	0.51	0.50	-0.87	0.62
CC[3] (22)	0.16	0.23	0.30	0.21	-0.69	-0.01	0.17	-0.67	0.03	-0.20	-1.00	-0.18
CC[5-I] (61)	0.37	0.45	0.55	-0.04	-0.70	-0.27	-0.12	-0.71	-0.27	-0.27	-0.92	-0.24
CC[5-J] (40)	0.63	0.65	0.77	-0.18	-0.81	-0.39	-0.23	-0.83	-0.37	-0.49	-0.98	-0.49
CD[6] (15)	0.67	0.72	0.83	0.78	0.00	0.72	0.80	-0.01	0.79	0.78	-0.39	0.80
LC[7] (164)	0.89	0.86	0.94	0.41	-0.55	0.28	0.42	-0.53	0.35	0.20	-0.85	0.25
NAFLD[8] (86)	0.65	0.65	0.75	0.29	-0.55	0.13	0.30	-0.53	0.23	0.18	-0.92	0.27
RA[9] (49)	0.56	0.57	0.66	0.38	-0.60	0.23	0.38	-0.59	0.29	0.57	-0.94	0.70

[a]Cliff's delta values for each healthy (column) and non-healthy (row) sub-cohort comparison across four different gut microbiome characteristics. [b]The sample size of each group or cohort is shown in parentheses. AS, ankylosing spondylitis; CA, colorectal adenoma; CC, colorectal cancer; CD, Crohn's disease, H, healthy; LC liver cirrhosis; NAFLD, non-alcoholic fatty liver disease; RA, rheumatoid arthritis. The number in superscript adjacent to phenotype abbreviations corresponds to a particular study used in validation. See **Supplementary Table 5** for study information.

SUPPLEMENTARY NOTE

Supplementary Note 1. Three major steps in the design of the Gut Microbiome Health Index (GMHI) for predicting health status.

1. For every possible pairwise combination of the prevalence fold-change threshold (θ_f) and the prevalence difference threshold (θ_d), the Health-prevalent (M_H) and Health-scarce (M_N) species that simultaneously satisfy both thresholds in the discovery cohort (i.e., training dataset composed of 4,347 stool metagenome samples) are obtained. Thus, each pair of thresholds leads to its respective set of M_H and M_N.
2. Then, each species set of M_H and M_N is used to find its 'collective abundance' in sample i ($\psi_{M_H,i}$ and $\psi_{M_N,i}$, respectively). In turn, h_{i,M_H,M_N}, which is the log-ratio of $\psi_{M_H,i}$ to $\psi_{M_N,i}$, is used to classify that sample i as healthy (i.e., $h_{i,M_H,M_N} > 0$), non-healthy (i.e., $h_{i,M_H,M_N} < 0$), or neither (i.e., $h_{i,M_H,M_N} = 0$). Accordingly, the balanced accuracy (χ_{M_H,M_N}), defined as the average of the proportions of 2,636 healthy and 1,711 non-healthy samples (all from our training dataset) that were correctly classified, is found. Thus, each pair of M_H and M_N species sets leads to its respective χ_{M_H,M_N}.
3. Finally, the classification model h_{i,M_H,M_N} (along with its inputs M_H and M_N) that results in the highest balanced accuracy χ_{M_H,M_N}^{max} on the discovery cohort is chosen as our final classifier.

Supplementary Note 2. Evaluation of other ecological characteristics in distinguishing healthy from non-healthy phenotypes of the validation cohort.

Opposite to its pattern from the discovery cohort, the Shannon diversities of the healthy validation group were slightly lower than those of the non-healthy validation group (two-sided Mann-Whitney *U* test, $P = 8.1\times10^{-3}$; Cliff's Delta = -0.16; **Supplementary Figure 9a**); this was also the case for 80% abundance coverage (two-sided Mann-Whitney *U* test, $P = 2.5\times10^{-2}$; Cliff's Delta = -0.13; **Supplementary Figure 9b**). On the other hand, species richness was found to be lower in the healthy validation group (Mann-Whitney *U* test, $P = 2.6\times10^{-10}$; Cliff's Delta = -0.37; **Supplementary Figure 9c**), which was consistent with our previous finding in the discovery cohort.

Across the individual sub-cohorts, Shannon diversity demonstrated a far less robust and consistent stratification between healthy and non-healthy compared to GMHI (**Supplementary Figure 9d**): the first healthy sub-cohort (H^1) was found to have significantly higher Shannon diversity than five disease sub-cohorts (AS, CA, CD, LC, and RA); the second healthy sub-cohort (H^2) was found to actually have significantly lower Shannon diversity than eight disease sub-cohorts (AS, CA, three sub-cohorts of CC, LC, NAFLD, and RA); and the third healthy sub-cohort (H^3) was found to have significantly higher Shannon diversity than only two disease sub-cohorts (AS and CD) (two-sided Mann-Whitney *U* test, $P < 0.01$; see **Supplementary Table 9** for Cliff's Deltas). Additionally, two healthy sub-cohorts were found to have significantly higher distributions of Shannon diversity than the third healthy sub-cohort (two-sided Mann-Whitney *U* test, $P < 0.01$), whereas no significant differences were found amongst all three healthy sub-cohorts for GMHI. Furthermore, the two highest Shannon diversities were observed in colorectal cancer sub-cohorts, whereas the highest GMHIs were observed in the three healthy sub-cohorts.

Finally, as was the case with Shannon diversity, we found very weak and inconsistent stratification between healthy and non-healthy sub-cohorts with 80% abundance coverage (**Supplementary Figure 9e**). H^1 had significantly higher distributions in five non-healthy sub-cohorts (AS, CA, CD, LC, and RA); H^2 had significantly lower distributions in seven non-healthy sub-cohorts (AS, three sub-cohorts of CC, LC, NAFLD, and RA); and H^3 had significantly higher distributions in four non-healthy sub-cohorts (AS, CA, CD, and LC)

(two-sided Mann-Whitney U test, $P < 0.01$; see **Supplementary Table 9** for Cliff's Deltas). Similarly, species richness also showed inconsistency in distinguishing healthy from non-healthy (**Supplementary Figure 9f**): H^1 had significantly higher distributions in three non-healthy sub-cohorts (CA, CD, and RA) and a lower distribution in one non-healthy sub-cohort (CC); H^2 had significantly lower distributions in seven non-healthy sub-cohorts (AS, three sub-cohorts of CC, LC, NAFLD, and RA); and H^3 had significantly higher distributions in three non-healthy sub-cohorts (CA, CD, and RA) but a lower distribution in one non-healthy sub-cohort (CC) (two-sided Mann-Whitney U test, $P < 0.01$; see **Supplementary Table 9** for Cliff's Deltas). Finally, as was the case for Shannon diversity, two healthy sub-cohorts were found to have significantly higher distributions of 80% abundance coverage and of species richness than a third healthy sub-cohort (two-sided Mann-Whitney U test, $P < 0.01$). In conclusion, GMHI is the most accurate, robust, and clinically meaningful classifier compared to other ecological characteristics.

SUPPLEMENTARY METHODS

Designing a classifier based upon Health-prevalent species to distinguish healthy and non-healthy groups. Since there are 7 microbial species identified as 'Health-prevalent', we classified each of the 4,347 metagenome samples in the training dataset as healthy if at least 1 of the 7 Health-prevalent species was present. This led to a balanced accuracy of 54.9%. Analogously, we classified each sample as healthy if at least 2 of the 7 Health-prevalent species were present (balanced accuracy: 61.3%). Continuing in an iterative manner, we obtained an balanced accuracy of 65.3%, 66.3%, 61.1%, 54.5%, and 51.4% when the minimally required count of present Health-prevalent species was set to 3, 4, 5, 6, and 7, respectively. Next, we used this approach on the 679 metagenome samples of the independent validation dataset; for this, we set 4 as the minimally required count of present Health-prevalent species (for a sample to be classified as healthy), as this threshold gave the best results with the training dataset. The balanced accuracy on the validation dataset resulted in 59.3%. All results are described in **Supplementary Table 6**. In stark contrast, GMHI displayed far better classification performance by achieving a balanced accuracy of 69.7% and 73.7% in the training and validation datasets, respectively.

Designing a classifier based upon Shannon Diversity to distinguish healthy and non-healthy groups. As Shannon diversity doesn't have a clear cut-off value to serve as a threshold for discriminating the two groups (in contrast, a sample with a positive and negative GMHI value is classified as healthy and non-healthy, respectively), we decided to apply three different thresholds and evaluate their performances separately: among the Shannon diversity measurements from all 4,347 samples of the training dataset, we selected: i) the 1st quartile (=2.50); ii) the median (=2.85); and iii) the 3rd quartile (=3.11). More specifically, any sample with a Shannon diversity equal to or greater than each threshold is classified as healthy; otherwise, as non-healthy. The balanced accuracy on the training dataset (4,347 samples) when using a threshold of Q1, median, and Q3 was found to be 52.9%, 53.6%, and 53.5%, respectively. Furthermore, on the independent validation dataset (679 samples), the balanced accuracy when using a threshold of Q1, median, and Q3 was found to be 43.0%, 47.0%, and 48.5%, respectively. All results are described in **Supplementary Table 7**.
