# Supplementary material for: A predictive index for health status using species-level gut microbiome profiling
Source: Nat Commun. 2020 Sep 15;11:4635. doi: 10.1038/s41467-020-18476-8 (PMC7492273; doi:10.1038/s41467-020-18476-8)
Supplement: Supplementary file 7 — Description of Additional Supplementary Files [file 41467_2020_18476_MOESM7_ESM.pdf]

Title: Supplementary Data 1
Description: Studies and samples used to construct a meta-dataset composed of 4,347 human stool metagenomes.

Title: Supplementary Data 2
Description: Taxonomic information of the 313 microbial species found to be present across 4,347 stool metagenomes.

Title: Supplementary Data 3
Description: Evidence from recent studies reporting associations between Health-prevalent/- scarce species and human health/disease.

Title: Supplementary Data 4
Description: Studies and samples used to construct the independent validation set composed of 679 human stool metagenomes
