# Supplementary material for: A predictive index for health status using species-level gut microbiome profiling
Source: Nat Commun. 2020 Sep 15;11:4635. doi: 10.1038/s41467-020-18476-8 (PMC7492273; doi:10.1038/s41467-020-18476-8)
Supplement: Supplementary file 8 — Reporting Summary [file 41467_2020_18476_MOESM8_ESM.pdf]

nature research

Corresponding author(s): Jaeyun Sung

Last updated by author(s): Aug 16, 2020

Reporting Summary

Nature Research wishes to improve the reproducibility of the work that we publish. This form provides structure for consistency and transparency in reporting. For further information on Nature Research policies, see our Editorial Policies and the Editorial Policy Checklist.

Statistics

For all statistical analyses, confirm that the following items are present in the figure legend, table legend, main text, or Methods section.

n/a	Confirmed	
☐	☒	The exact sample size (n) for each experimental group/condition, given as a discrete number and unit of measurement
☐	☒	A statement on whether measurements were taken from distinct samples or whether the same sample was measured repeatedly
☐	☒	The statistical test(s) used AND whether they are one- or two-sided<br>*Only common tests should be described solely by name; describe more complex techniques in the Methods section.*
☐	☒	A description of all covariates tested
☐	☒	A description of any assumptions or corrections, such as tests of normality and adjustment for multiple comparisons
☐	☒	A full description of the statistical parameters including central tendency (e.g. means) or other basic estimates (e.g. regression coefficient) AND variation (e.g. standard deviation) or associated estimates of uncertainty (e.g. confidence intervals)
☐	☒	For null hypothesis testing, the test statistic (e.g. F, t, r) with confidence intervals, effect sizes, degrees of freedom and P value noted<br>*Give P values as exact values whenever suitable.*
☒	☐	For Bayesian analysis, information on the choice of priors and Markov chain Monte Carlo settings
☒	☐	For hierarchical and complex designs, identification of the appropriate level for tests and full reporting of outcomes
☐	☒	Estimates of effect sizes (e.g. Cohen's d, Pearson's r), indicating how they were calculated

Our web collection on statistics for biologists contains articles on many of the points above.

Software and code

Policy information about availability of computer code

Data collection	Software for shotgun metagenomic sequencing:<br>The flow cells were sequenced as 150 x 2 paired-end reads on an Illumina HiSeq 4000 using the HiSeq 3000/4000 sequencing kit and HiSeq Control Software HD 3.4.0.38. Base-calling was performed using Illumina’s RTA version 2.7.7.

Data analysis

Quality control of sequencing data:
Sequence reads were processed with the KneadData v0.5.1 quality-control pipeline (http://huttenhower.sph.harvard.edu/kneaddata), which uses Trimmomatic v0.36 and Bowtie2 v0.1 for removal of low-quality read bases and human reads, respectively. Trimmomatic v0.36 was run with parameters SLIDINGWINDOW:4:30, and Phred quality scores were thresholded at '<30'. Illumina adapter sequences were removed, and trimmed non-human reads shorter than 60 bp in nucleotide length were discarded. Potential human contamination was filtered by removing reads that aligned to the human genome (reference genome hg19).

Analysis of shotgun metagenomic sequencing data:
Phylogenetic clade and MetaCyc pathway abundance profiling was done using the MetaPhlAn2 v2.7.0 and HUMAnN2 pipeline, respectively.

Statistical analyses:
R v4.0.0 was used for all statistical analyses. The R packages 'ade4' version 1.7-15 and 'vegan' version 2.5.6 were used to perform Principal Coordinate Analysis (PCoA) ordination with Bray-Curtis dissimilarity. The R package 'vegan' was used to calculate Shannon diversity (Shannon index) and species richness based on the species abundance profiles for each sample of our meta-dataset. A mixed-effects linear regression model was used to investigate the statistical association between GMHI and library size ('lmer' function in the R package 'lme4' version 1.1-23). A classifier based upon a Random Forests algorithm was designed and (data) curation performed in Python version 3.6.4., while model implementation was performed in the 'scikit-learn' Python package version 0.23.1. R scripts demonstrating how to reproduce all of our findings shown in the main figures, as well as how to calculate GMHI for a given stool metagenome sample, are available at https://github.com/jaeyunsung/GMHI_2020.

For manuscripts utilizing custom algorithms or software that are central to the research but not yet described in published literature, software must be made available to editors and reviewers. We strongly encourage code deposition in a community repository (e.g. GitHub). See the Nature Research guidelines for submitting code & software for further information.

Data

Policy information about availability of data

All manuscripts must include a data availability statement. This statement should provide the following information, where applicable:
- Accession codes, unique identifiers, or web links for publicly available datasets
- A list of figures that have associated raw data
- A description of any restrictions on data availability

Data availability: Raw sequencing data accession IDs of all publicly available stool metagenome samples (and their corresponding studies) used in all analyses of this study are available in Supplementary Data 1 and Supplementary Data 4. Sequences for the dataset containing rheumatoid arthritis stool metagenomes used for GMHI validation have been deposited at NCBI's Sequence Read Archive (SRA) data repository (BioProject number PRJNA598446; https://www.ncbi.nlm.nih.gov/sra/?term=PRJNA598446), and can be downloaded without any restrictions. The deposited sequences include .fastq files for 49 patients with rheumatoid arthritis. Measurements were taken from distinct samples. Human reads were identified and removed prior to data upload.

Field-specific reporting

Please select the one below that is the best fit for your research. If you are not sure, read the appropriate sections before making your selection.

[x] Life sciences   [ ] Behavioural & social sciences   [ ] Ecological, evolutionary & environmental sciences

For a reference copy of the document with all sections, see nature.com/documents/nr-reporting-summary-flat.pdf

Life sciences study design

All studies must disclose on these points even when the disclosure is negative.

Sample size

No sample-size calculation was performed prior to the initiation of the computational analyses. For the training data, we collected 4,347 stool metagenome samples, which was as many publicly available samples as we could possibly find. All statistical analyses between the healthy and non-healthy groups came out to be significant, thereby justifying that these sample sizes were sufficient.

Data exclusions	Quality filtration of metagenome studies and samples was done at the time of raw data processing based upon the following pre-established criteria mentioned in the Methods section and in Fig. 1a of the manuscript:<br>- In studies wherein multiple samples were taken per individual across different time-points, we included only the first or baseline sample in the original study.<br>- We excluded studies pertaining to diet or medication interventions (to avoid the chances of unnatural outcomes due to effects of interventions on gut microbiome), or those with fewer than 10 samples.<br>- Samples from subjects who were less than 10 years of age were also excluded from our analysis.<br>- Samples that were collected from disease controls, but were not reported as healthy nor had any mentioning of diagnosed disease in the original study, were excluded from our analysis.<br>- Sample-filtering based on taxonomic profiles. After taxonomic profiling, the following stool metagenome samples were discarded from our analysis: i) samples composed of more than 5% unclassified taxonomies (100 samples); and ii) phenotypic outliers according to a dissimilarity measure. More specifically, Bray-Curtis distances were calculated between each sample of a particular phenotype and a hypothetical sample in which the species' abundances were taken from the medians across those samples. A sample was considered as an outlier, and thereby removed from further analysis, when its dissimilarity exceeded the upper and inner fence (i.e., > 1.5 times outside of the interquartile range above the upper quartile and below the lower quartile) amongst all dissimilarities.<br><br>No metagenome studies or samples were excluded beyond these pre-established criteria.
Replication	An independent external validation dataset, composed of 679 stool metagenome samples, was used to confirm the reproducibility of our prediction results in stratifying healthy and non-healthy groups. This validation dataset was not part of the training dataset used for the original formulation of GMHI.
Randomization	Stool metagenome samples from published studies were allocated into one of two groups based on how they had been described in their original studies: healthy (i.e., absence of a diagnosed disease) or non-healthy (i.e., presence of a diagnosed disease). No randomization was required, because our study is a cross-sectional analysis of published datasets and NOT a clinical trial. An unbiased search was performed while collecting microbiome samples from public repositories. Study IDs were included in the PERMANOVA analysis to control for possible study-specific confounding effects.
Blinding	Blinding to group allocation is not relevant to our bioinformatics-based, cross-sectional study. We were not blinded to subject group, as knowing the subject group (healthy or non-healthy) of each microbiome sample was critical for achieving the goals of this study. To further remove any source of possible bias, we had all stool metagenome samples analyzed simultaneously on one computer by one single person and later confirmed by another.

Reporting for specific materials, systems and methods

We require information from authors about some types of materials, experimental systems and methods used in many studies. Here, indicate whether each material, system or method listed is relevant to your study. If you are not sure if a list item applies to your research, read the appropriate section before selecting a response.

Materials & experimental systems

n/a	Involved in the study
☒	☐ Antibodies
☒	☐ Eukaryotic cell lines
☒	☐ Palaeontology and archaeology
☒	☐ Animals and other organisms
☐	☒ Human research participants
☒	☐ Clinical data
☒	☐ Dual use research of concern

Methods

n/a	Involved in the study
☒	☐ ChIP-seq
☒	☐ Flow cytometry
☒	☐ MRI-based neuroimaging

Human research participants

Policy information about studies involving human research participants

Population characteristics	Stool samples that were already preserved in the Mayo Clinic Rheumatology Biobank were obtained from 49 patients with rheumatoid arthritis. Mean age: 65.1; Sex (M/F/NA): 14/29/6; Geographical origin: USA; Diagnosis at time of sample collection: Rheumatoid arthritis with varying stages of disease activity; Treatment categories: mixture of bDMARDs, csDMARDs, and Prednisone.
Recruitment	Patients seeking treatment for rheumatoid arthritis volunteered to donate their stool samples to the Mayo Clinic Rheumatology Biobank. No preference regarding age, sex, geography was considered; this is deemed unlikely to impact the main study conclusions, given the highly randomized nature of sample covariates, as well as the overall study design.
Ethics oversight	All stool samples were obtained following written informed consent. All samples were de-identified prior to processing for sequencing and data analysis. The collection of biospecimens was approved by the Mayo Clinic Institutional Review Board (#14-000616).

Note that full information on the approval of the study protocol must also be provided in the manuscript.
